# Prognostic analysis of endovascular mechanical thrombectomy in stroke patients with acute internal carotid artery obstruction based on circle of Willis variation

**DOI:** 10.3389/fneur.2024.1428721

**Published:** 2025-01-07

**Authors:** Tianlun Qiu, Huagang Luo, Wuqiao Bao

**Affiliations:** Department of Neurosurgery, Shaoxing People's Hospital, Shaoxing, China

**Keywords:** acute intracranial internal carotid artery occlusion, Willis compensation, collateral circulation, circle of Willis, prognosis

## Abstract

**Objective:**

Endovascular mechanical thrombectomy (EVMT) is widely employed in patients with acute intracranial carotid artery occlusion (AIICAO). This study aimed to predict the outcomes of EVMT following AIICAO by utilizing anatomic classification of the circle of Willis and its relative position to the thrombus.

**Methods:**

In this study, we retrospectively analyzed a cohort of 108 patients with AIICAO who underwent endovascular mechanical thrombectomy (EVMT) at Shaoxing People’s Hospital. Based on variations in the circle of Willis, as well as the size and location of the thrombus occluding the middle cerebral artery (MCA), anterior cerebral artery (ACA), and posterior cerebral artery (PCA), we classified AIICAO into four grades using digital subtraction angiography (DSA). EVMT was initiated upon admission, and baseline data including demographic characteristics, vascular risk factors, angiographic features, initial National Institutes of Health Stroke Scale (NIHSS) scores, Alberta Stroke Program Early CT Score (ASPECT), and etiology classification were compared across these four grades. The prognosis and mortality rates at 90 days post-stroke were evaluated for the different grades and within each grade, patients were further categorized into two subtypes based on vascular compensation and occluded vessels.

**Results:**

Significant differences were observed among the four grades of Willis compensation concerning etiologic classification (*p* = 0.008), postoperative modified treatment in cerebral ischemia (mTICI, *p* = 0.017), postoperative symptomatic intracranial hemorrhage (sICH, *p* = 0.007), NIHSS score at admission (*p* = 0.001), and favorable outcomes at 90 days (modified Rankin Score 0–2) (*p* = 0.003). The mortality rate at 90 days exhibited a significant difference across the four grades of Willis compensation (*p* = 0.05). However, prognosis did not reveal any significant differences among the various subtypes within the same grade (*p* > 0.05).

**Conclusion:**

The assessment of the degree of Willis compensation can be improved by evaluating the integrity of the circle of Willis, as well as the size and location of the clot in cases of isolated internal carotid artery occlusion (iICAo). This approach provides valuable prognostic indicators and important insights for the pre-selection of patients prior to endovascular mechanical thrombectomy (EVMT).

## Introduction

Acute ischemic stroke (AIS) resulting from the occlusion of a large vessel is a critical condition that can lead to sudden death. However, endovascular mechanical thrombectomy (EVMT) has emerged as a significant treatment modality for AIS patients with large vessel occlusions. It is well-established that variations in occlusion sites can lead to diverse symptoms and prognoses, and among these, AIICAO is associated with a higher mortality rate.

The collateral circulation provided by the Willis collaterals and the leptomeningeal collaterals (LMC) is essential for protecting the ischemic penumbra and extending the time variable for EVMT (1, 2). The circle of Willis is plays a critical role in maintaining stable intracranial blood flow and perfusion pressure (3, 4). However, considerable morphological variations in the Willis structure in the general population, with fewer than 50% of individuals exhibiting a complete Willis circle (5, 6). These variations can compromise the compensatory function of the Willis structure in regulating cerebral arterial blood flow. Consequently, it is important to distinguish the collateral blood flow patterns associated with different configurations of the Willis circles and thrombus causing ICA occlusion, which can potentially impact revascularization, ischemic injury, and other associated clinical outcomes (6–8). To tackle this issue, the patterns of ICA occlusion patterns have been subclassified into I-type, L-type, and T-type, these classifications depend on the patency and blood flow of the ipsilateral intracranial artery (9–11).

However, the above methods did not systematically compare the various anatomical variations and thrombus localizations of Willis. Evaluation of blood supply to the brain tissue through the branches of the internal carotid artery (ACA, MCA, and PCA). Considering the anatomical structure of the circle of Willis and the location of the thrombus is an accurate approach for analyzing the volume of brain tissue ischemia for predicting prognosis of EVMT in AIICAO patients. Our analysis focused on the different anatomic variations of the circle of Willis and the effect of various thrombus localizations on the blood supply to the branches of the internal carotid artery.

## Materials and methods

### Patient data and protocol

**Figure 1:**
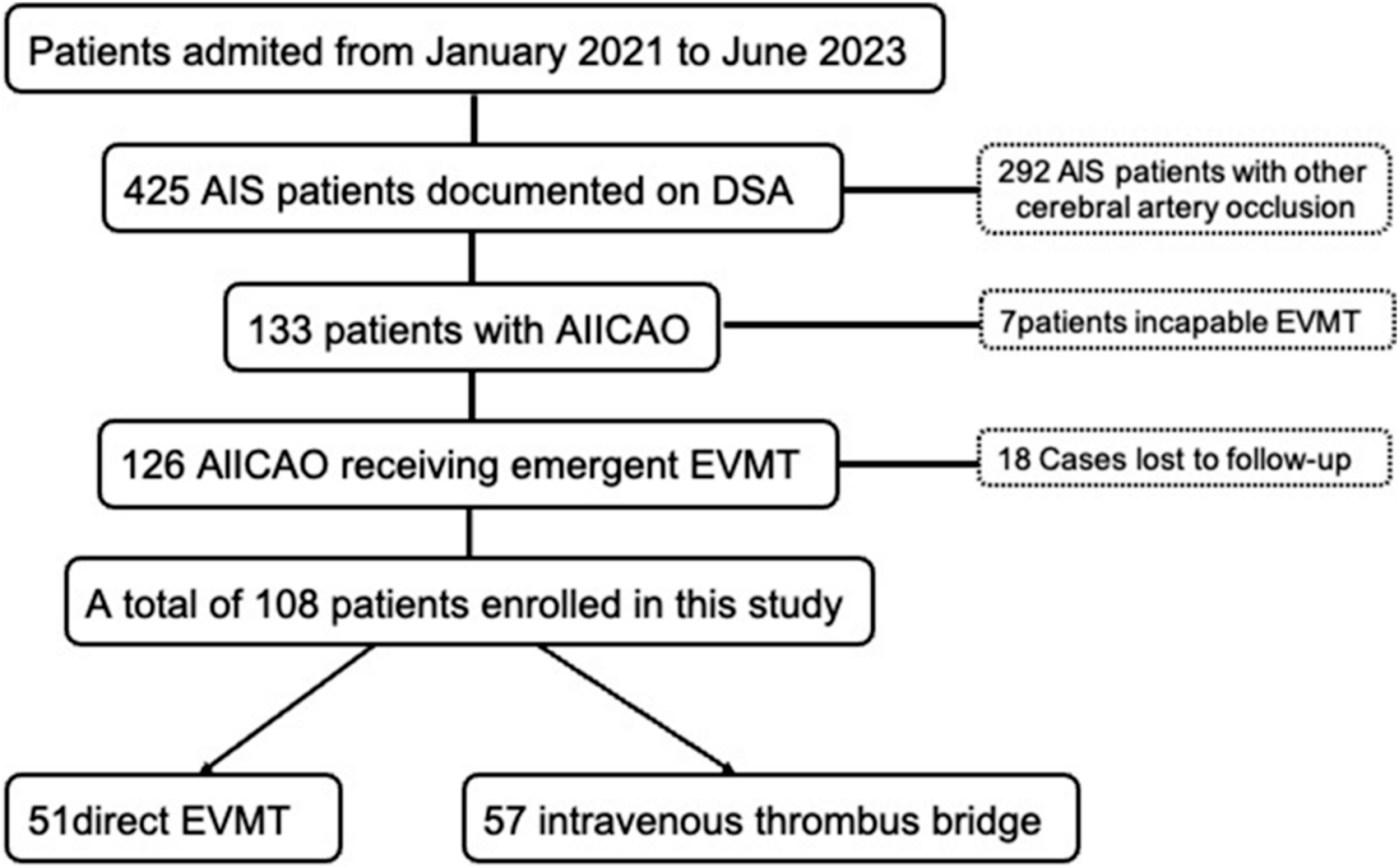
Flow chart of the patients in this study.

In this retrospective study, we examined patients with acute ischemic stroke at Shaoxing People’s Hospital in Zhejiang Province, China, from January 2021 to June 2023. We enrolled patients diagnosed with AIICAO who received urgent EVMT. Initially, patients eligible for treatment with intravenous recombinant tissue plasminogen activator (rt-PA) within 4.5 h of stroke were first treated with intravenous thrombolysis and subsequently transferred to interventional therapy; other patients received emergency EVMT exclusively. The study included patients who met the following criteria: acute intracranial carotid artery occlusion, modified Rankin Scale (mRS) pre-stroke score of less than 2 points, National Institutes of Health Stroke Scale (NIHSS score) ≥6; and no large low-density shadow (Alberta Stroke Program Early CT Score [ASPECT] ≥ 6) observed on CT within 6 h of stroke onset. Patients with symptomatic intracranial hemorrhage (SICH) or severe ischemic infarction (defined as acute ischemic changes affecting more than one-third of the middle cerebral artery or more than 100 mL of tissue in other areas) evidenced by CT or MRI were excluded from the study. All included patients were treated under general anesthesia. Digital cerebral subtraction angiography of the aortic arch was manipulated to identify collateral circulation, including the integrity of the circle of Willis and the pial collaterals. A total of 425 AIS cases were treated in the hospital, comprising 148 cases of MCA trunk occlusion, 77 cases of MCA branch occlusion, 16 cases of ACA occlusion, 51 cases of vertebrobasilar artery occlusion, and 133 cases of ICA occlusion. Among the 133 patients with ICA occlusion, 7 patients could not undergo EVMT for various reasons and 18 patients lost to follow-up. EVMT was performed directly in 51 patients, while bridging therapy with intravenous thrombolysis was administered to 57 patients (Figure 1). This study received approval from the Ethics Committee of Shaoxing People’s Hospital (No. 2023-073-01).

### Classification of AIICAO occlusion

**Figure 2:**
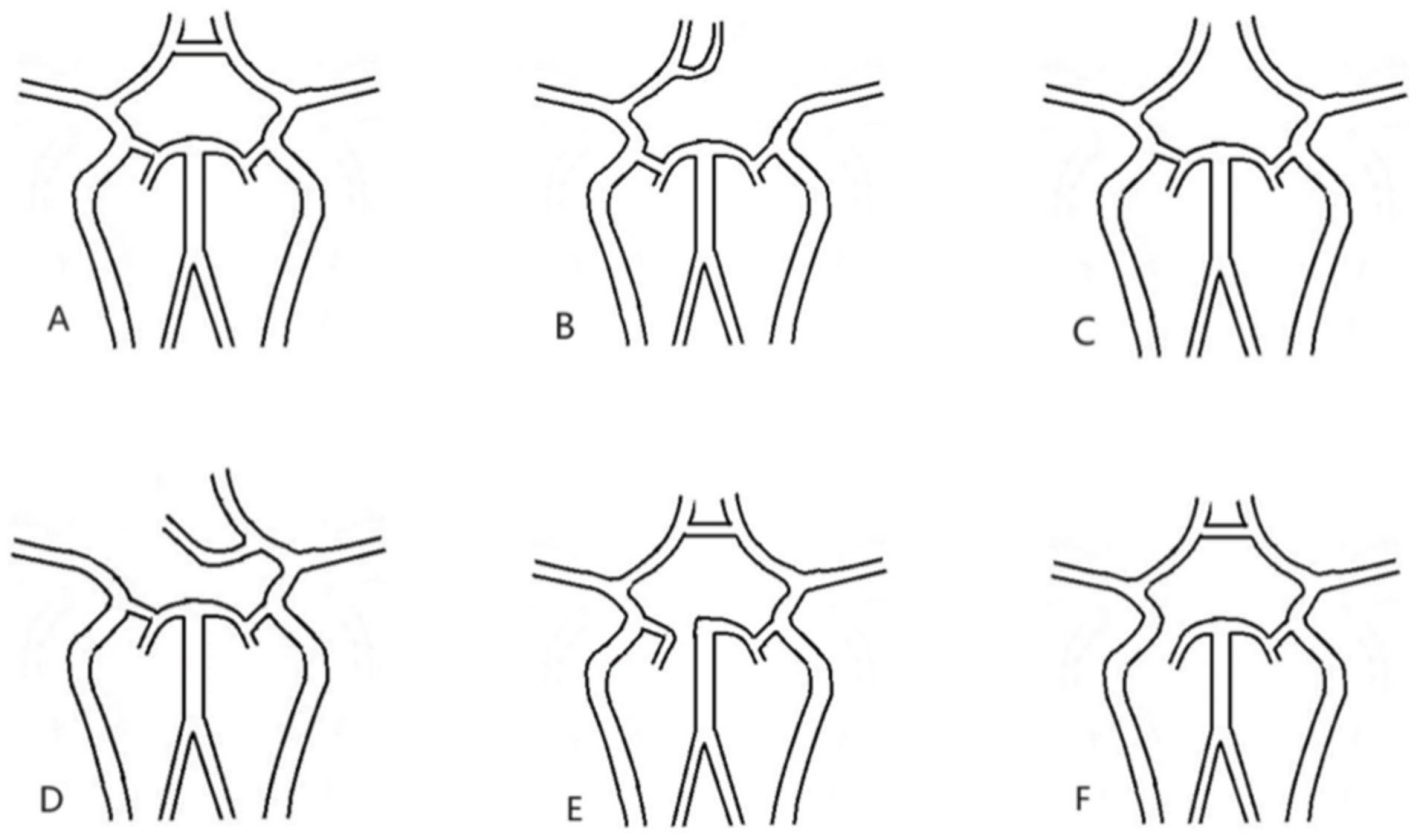
All types of Willis. **(A)** Intact willis, **(B)** lacking contralateral A1, **(C)** lacking ACoA, **(D)** lacking ipsilateral A1, **(E)**. lacking P1, **(F)**. lacking PCoA. ACoA: anterior communicating artery; PCoA: posterior communicating artery.

There is a large of literatures exists regarding the anatomical variation of Willis (12–14). The prognosis of AIICAO patients following EVMT was evaluated base on the anatomic variation of Willis, the different positions of the thrombus in Willis, the size and shape of the thrombus. According to the literatures, the morphology of the Willis is categorized into six types, including (A) Intact Willis, (B) Absence of contralateral anterior cerebral artery (ACA), (C) Absence of anterior communicating artery (ACoA), (D) Absence of internal carotid artery (ICA), (E) Absence of posterior cerebral artery (PCA), (F) Absence of posterior communicating artery (PCoA) as illustrated in this study (Figure 2).

**Figure 3:**
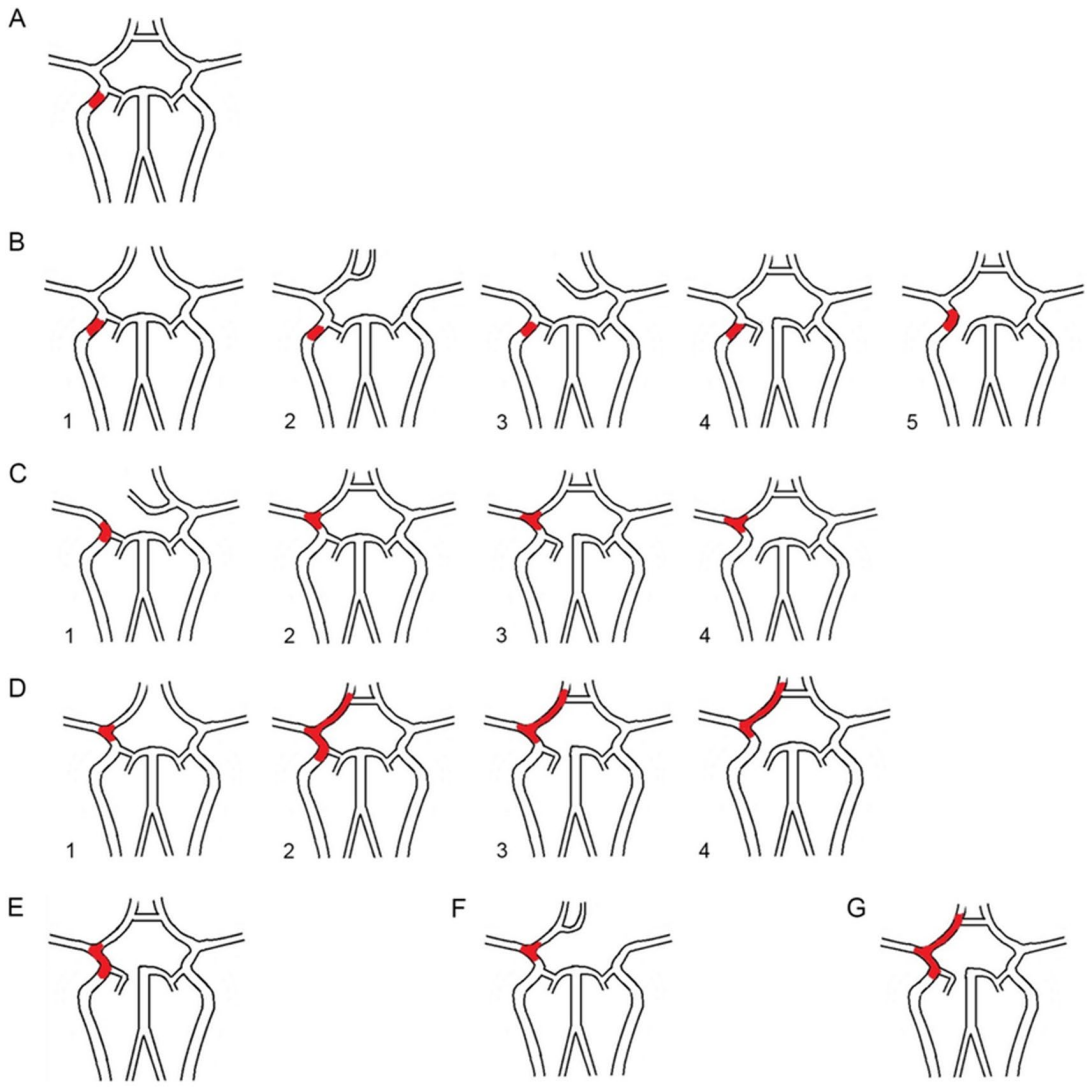
**(A)** The proximal ICA occlusion of type 1. **(B1)**. The dual supply of PCoA and ACoA, **(B2–5)**: only PCoA or ACoA supply; **(C1-4)**. The ICA + MCA occlusion of type 2; **(D1-4)**. The ICA + MCA + ACA of type 3; **(E)**. The ICA + MCA+ PCA of type 4; **(F)**. The ICA + MCA+ 2ACA occlusion of type5; G. The ICA + MCA+ ACA + PCA occlusion of type6. ICA: internal carotid artery; MCA: middle cerebral artery; ACA: anterior cerebral artery; PCA: posterior cerebral artery.

**Figure 4:**
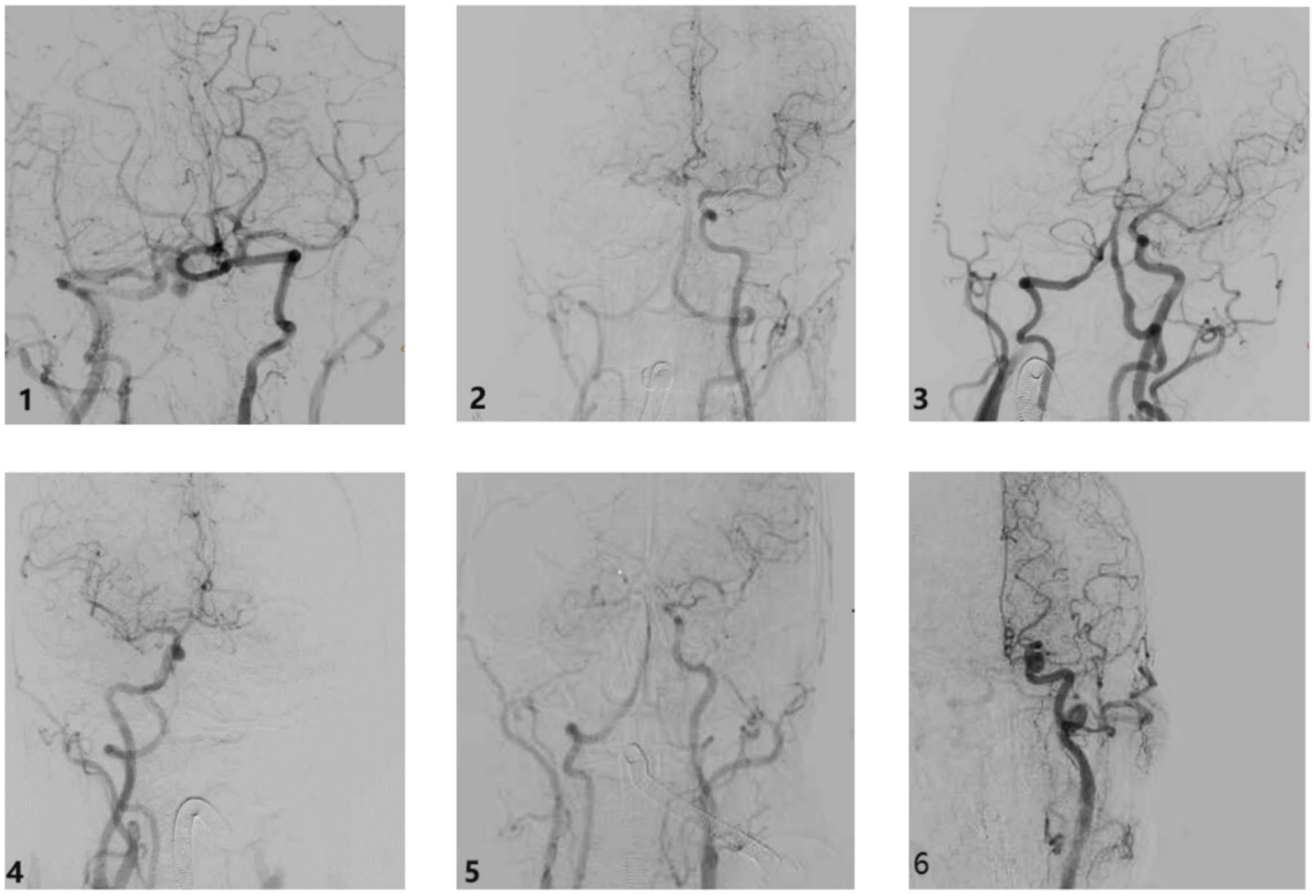
The example of types in aortic arch angiography ICA occlusion type. Type 1: ICA occlusion, Type 2: ICA + MCA occlusion, Type 3: ICA + MCA + ACA occlusion, Type 4: ICA + MCA + PCA occlusion, Type 5: ICA + MCA + 2ACA occlusion, Type 6: ICA + MCA + PCA occlusion. ICA: internal carotid artery; MCA: middle cerebral artery; ACA: anterior cerebral artery; PCA: posterior cerebral artery.

Based on the occlusion position and the length of the thrombus across these six types, we examined the involvement of each major cerebral artery. We categorized the AIICAO patients into seven types of Willis compensation (Model 1) including type 1 (proximal ICA occlusion, with subtypes 1a: dual supply from PCoA and ACoA, and type 1b: only PCoA or ACoA supply) (Figures 3A,B), type 2 (ICA + MCA closure) (Figure 3C), type 3 (ICA + MCA + ACA occlusion) (Figure 3D), type 4 (ICA + MCA + PCA occlusion) (Figure 3E), type 5 (ICA + MCA+ 2ACA occlusion) (Figure 3F), type 6 (ICA + MCA+ ACA + PCA occlusion) (Figure 3G). Example of types in aortic arch angiography are shown for ICA occlusion type 1–6 (Figure 4).

To streamline the clinical prognosis, the seven types of cases were statistically analyzed and categorized into four grades (Model 2), the first grade includes of types 1a and 1b, the second grade encompasses type 2, the third grade includes type 3 and 4, and the fourth grade comprises types 5 and 6.

### Data collection

#### Demographic data

The neurological status of each patient was assessed upon admission and discharge using the National Institute of Health Stroke Scale (NIHSS) score (15, 16). Patients who died were assigned an NIHSS score of 42 (17).

The following information from the patients were collected through the hospital database: age, sex, diabetes mellitus, hypertension, coronary artery disease, anticoagulants, intravenous thrombolysis, ASPECT score at admission, NIHSS score on admission, and the etiologic subtype of thrombus (including atherosclerosis *in situ*, cardioembolism, decadent atherothrombotic plaque and undetermined etiology) (13).

#### Prognosis data

The degree of reperfusion was quantified according to the modified classification of thrombolysis in cerebral infarction (mTICI) (18). Recanalization was assessed based on the TICI score and defined as successful (mTICI 2B or 3) and unsuccessful (mTICI 0/1/2A) (19). The prognosis was considered favorable if a patient achieved a Modified Rankin Scale score of 0–2 at three months, during which the three mortality rate was also analyzed.

Patients’ clinical outcomes were collected from a specialized database dedicated to stroke prevention and management in China, which prospectively collected the modified Rankin Scale (mRS) to improve the prognosis. To evaluate survival, the patients were followed up as outpatients or through telephone contact 90 days post-surgery, and the prognosis was assessed using the mRS Scale. Patients were defined as favorable outcome (mRS ≤ 2) and unfavorable outcome (mRS ≥ 3) at 90 days. Additionally, the postoperative mTICI scores and occurrences of spontaneous intracerebral haemorrrhage (sICH) were documented.

### Statistical analysis

The SPSS software for Windows (version 26.0, IBM., NY, United States) was utilized for statistical analysis. Numerical variables were presented as mean ± standard deviation (SD), and compared using analysis of variance. Categorical variables were expressed as n (%), and compared by chi-square test analysis. Factors among the 7 types of Willis compensation (Model 1) were compared by chi-square test and analysis of variance. There were significant differences in the etiologic and prognostic data across the 7 type groups. The etiologic and prognostic data for type 1a and type 1b, type 3 and type 4, as well as type 5 and type 6 were analyzed separately. If no significant differences were identified, these groups were classified into grade 1 to grade 4 (Model 2), and followed by a statistical analysis of the etiologic and prognostic data.

## Results

A total of 425 AIS patients were registered, of which 317 patients were excluded (Figure 1). Table 1 presents the demographic data categorized by the seven types of occlusion (Model 1). Based on occlusion patterns the patients were classified into different subclasses: type 1a (15 patients), type 1b (5 patients), type 2 (53 patients), type 3 (16 patients), type 4 (7 patients), type 5 (9 patients), and type 6 (3 patients). There were no significant differences in sex distribution, age, history of diabetes mellitus, hypertension, coronary artery disease, anticoagulants, intravenous thrombolysis, and ASPECT at admission. The etiological subtype of stroke varied among the seven types of Willis compensation (*p* = 0.022, Table 1). Postoperative data indicated that the mTICI was significantly different across these seven types of Willis compensation (*p* = 0.04, Table 2). NIHSS scores at admission showed significant variation among the seven types of Willis compensation (*p* = 0.001, Table 2). Specifically, type 1a exhibited significantly lower than all other types (*p* < 0.05, Table 2), while type 2 was significantly lower than type 5 and type 6 (*p* < 0.05, Table 2). Furthermore, the 90 day outcomes, as measured by the modified Rankin Score (0–2), varied among the seven types of Willis compensation (*p* = 0.003, Table 2). Postoperative sICH also differed among the seven types of Willis compensation (*p* = 0.032, Table 2). There was no significant difference found in etiological and prognostic data of type1a and type1b, type3 and type4, type5 and type6 (*p* > 0.05, Table 3). Stroke etiological subtype, post-operative mTICI, postoperative sICH and 90 days favorable outcomes for the 4 grades of Willis compensation (model 2) were significantly different (*p* < 0.05, Table 4). Mortality rates after 90 days of Willis compensation also varied significantly (*p* = 0.05, Table 4). The presence of deciduous atherothrombotic plaque in grade 4 was greater than in the other grades (*p* < 0.05, Table 4). Post-operative mTICI in grade 4 were lower compared to the in other grades (*p* < 0.05, Table 4). NIHSS score on admission for grade 1 was significantly lower than that for grade 2, grade 3 and grade 4, while grade 2 was significantly lower than grade 4 and higher than grade 1(*p* < 0.05, Table 4). Furthermore, the proportion of favorable outcomes at 90 days for grades 3 and 4 was significantly lower than that for grades 1 and 2 (Table 4).

**Table 1:** Univariate analysis of thrombectomy data among the 7 groups of Willis compensation in acute intracranial internal carotid artery oclusion(model 1).

Thrombectomy parameters	All	Type 1(proximal ICA occlusion)		Type 2 (ICA + MCA occlusion)	Type 3(ICA + MCA + ACA occlusion)	Type 4 (ICA + MCA + PCA occlusion)	Type 5 (ICA + MCA+ 2ACA occlusion)	Type 6 (ICA + MCA+ ACA + PCA occlusion)	*p* value
		**1**a: dual supply of PCoA and ACoA	1b: only PCoA or ACoA supply						
Number	108	15	5	53	16	7	9	3	
Sex									0.313#
Male(%)	75(69.4)	13(86.7)	3(60)	39(73.6)	9(56.3)	4(57.1)	6(66.7)	1(33.3)	
Female(%)	33(30.6)	2(13.3)	2(40)	14(26.4)	7(43.8)	3(42.9)	3(33.3)	2(66.7)	
Mean age(year)	70.5 ± 11.8	69.3 ± 13.5	68.2 ± 11.7	70.1 ± 11.8	70.0 ± 14.2	74.7 ± 6.2	74.9 ± 10.2	67.7 ± 9.9	0.849^
ASPECT on admission	9.21 ± 1.27	9.13 ± 1.25	9.20 ± 1.09	9.38 ± 1.18	9.13 ± 1.46	9.57 ± 1.13	8.33 ± 1.50	9.00 ± 1.73	0.430^
History of anticoagulation	20(18.5)	4(26.7)	1(20)	6(11.3)	5(31.3)	1(14.3)	2(22.2)	1(33.3)	0.584#
History of coronary artery disease(%)	33(30.8)	4(26.7)	2(40)	17(32.7)	4(25)	2(28.6)	3(33.3)	1(33.3)	0.995#
History of Diabetes Mellitus(%)	28(25.9)	4(26.7)	1(20)	14(26.4)	6(37.5)	2(28.6)	1(11.1)	0(0)	0.855#
History of hypertension(%)	77(71.3)	7(46.7)	4(80)	38(71.7)	13(81.3)	5(71.4)	8(88.9)	2(66.7)	0.355#
Stroke etiological subtype									0.022#
Atherosclerosis in situ(%)	32(29.6)	8(53.3)	3(60)	12(22.6)	6(37.5)	0(0)	2(22.2)	1(33.3)	
Cardioembolism(%)	51(47.2)	3(20)	1(20)	30(56.6)	7(43.8)	6(85.7)	3(33.3)	1(33.3)	
Undetermined etiology(%)	12(11.1)	1(6.7)	0	8(15.1)	2(12.5)	1(14.3)	0(0)	0(0)	
Deciduous atherothrombotic plaque (%)	13(12.0)	3(20)	1(20)	3(5.7)	1(6.3)	0(0)	4(44.4)	(33.3)	
Intravenous thrombolysis	51(47.2)	7(46.7)	1(20)	22(41.5)	10(62.5)	4(57.1)	6(66.7)	1(33.3)	0.537#

#Fisher exact probability method. ^ variance test. ASPECT, Alberta Stroke Program Early CT Score; PCoA, posterior communicating artery; ACoA, anterior communicating artery; ACA, anterior cerebral artery; PCA, posterior cerebral artery; MCA, middle cerebral artery; ICA, internal carotid artery.

**Table 2:** Univariate analysis of thrombectomy data among the 7 groups of Willis compensation in acute intracranial internal carotid artery occlusion (model 1).

Thrombectomy parameters	All	Type1(proximal ICA occlusion)		Type 2 (ICA + MCA occlusion)	Type 3 (ICA + MCA + ACA occlusion)	Type 4 (ICA + MCA + PCA occlusion)	Type 5 (ICA + MCA+ 2ACA occlusion)	Type 6 (ICA + MCA+ ACA + PCA occlusion)	*P* value
		1a: dual supply of PCoA and ACoA	1b: only PCoA or ACoA supply						
Number	108	15	5	53	16	7	9	3	
Post-operative mTICI									0.04#
0,1 or 2a(%)	19(17.6)	2(13.3)	1(20)	6(11.3)	4(25)	0(0)	4(44.4)	2(66.7)	
2b or 3(%)	89(82.4)	13(86.7)	4(80)	47(88.7)	12(75)	7(100)	5(55.6)	1(33.3)	
Postoperative sICH(%)	31(28.7)	2(13.3)	0(0)	13(24.5)	7(43.8)	3(42.9)	6(66.7)	0(0)	0.032#
NIHSS score on admission	17.5 ± 5.37	12.4 ± 5.15*	17.0 ± 7.75	17.2 ± 4.96&	18.8 ± 3.66	20.0 ± 5.03	22.0 ± 3.74	22.3 ± 1.53	0.001
90-Day good outcome (modified Rankin Score 0–2), n (%)	46(42.6)	10(66.7)a	4(80)ab	27(50.9)ab	2(12.5)b	1(14.3)ab	2(22.2)ab	0(0)ab	0.003
Motality after 90-Day(%)	29(26.9)	1(6.7)	0(0)	14(26.4)	7(43.8)	3(42.9)	3(33.3)	1(33.3)	0.159#

#Fisher exact probability method. *Type1a was significant lower than all other types (*P* < 0.05), type 2 was significantly lower than type 5 and type 6 (*p* < 0.05). a and b mean significantly different level in each group in outcome (*p* < 0.05). mTICI, modified Thrombolysis in Cerebral Infarction classification; SICH, Symptomatic intracranial hemorrhage; NIHSS, National Institute of Health Stroke Scale; ACA, anterior cerebral artery; PCA, posterior cerebral artery; MCA, middle cerebral artery; ICA, internal carotid artery; mRS, modified Rankin Score.

**Table 3:** Univariate analysis of thrombectomy data between 3 pairs of the type 1a and 1b, type3 and type 4, type5 and type 6 groups of Willis compensation in acute intracranial internal carotid artery occlusion.

Thrombectomy parameters	*P* value		
	Type 1a: dual supply of PCoA and ACoA Type 1b: only PCoA or ACoA supply	Type 3(ICA + MCA + ACA occlusion) type 4 (ICA + MCA + PCA occlusion)	Type 5 (ICA + MCA+ 2ACA occlusion) type 6 (ICA + MCA+ ACA + PCA occlusion)
Stroke etiological subtype	1.000	0.217	1.000
Post-operative mTICI	0.601	0.273	1.000
0,1 or 2a(%)			
2b or 3(%)			
Postoperative sICH(%)	1.000	1.000	0.182
NIHSS score on admission	0.427	0.819	
90-Day good outcome (modified Rankin Score 0–2), n (%)	1.000	1.000	1.000
Motality after 90-Day(%)	1.000	1.000	1.000

mTICI, modified Thrombolysis in Cerebral Infarction classification; SICH, Symptomatic intracranial hemorrhage; NIHSS, National Institute of Health Stroke Scale; ACA, anterior cerebral artery; PCA, posterior cerebral artery; MCA, middle cerebral artery; ICA, internal carotid artery; mRS, modified Rankin Score.

**Table 4:** Univariate analysis of thrombectomy data among the 4 grades of Willis compensation in acute intracranial internal carotid artery occlusion (model 2).

Thrombectomy parameters	All	Grade 1(proximal ICA occlusion)	Grade 2(ICA + MCA occlusion)	Grade 3 (ICA + MCA + ACA /PCA occlusion)	Grade 4 (ICA + MCA+ 2ACA/ ACA + PCA occlusion)	*P* value
Number	108	18	53	25	12	
Stroke etiological subtype						0.008#
Atherosclerosis in situ(%)	32(29.6)	9(50)	12(22.6)	8(32)	3(25)	
Cardioembolism(%)	51(47.2)	4(22.2)	30(56.6)	13(52.0)	4(33.3)	
Undetermined etiology(%)	12(11.1)	1(5.6)	8(15.1)	3(12.0)	0(0)	
Deciduous atherothrombotic plaque (%)	13(12.0)ab	4(22.2)b	3(5.7)b	1(4.0)b	5(41.7)a	
Post-operative mTICI						0.017
0,1 or 2a(%)	19(17.6)	3(16.7)ab	6(11.3)b	4(16.0)ab	6(50)a	
2b or 3(%)	89(82.4)	15(83.3)ab	47(88.7)b	21(84.0)ab	6(50)a	
Postoperative sICH(%)	31(28.37)	2(11.1)	13(24.5)	10(40)	6(50)	0.007
NIHSS score on admission	17.47 ± 5.37	13.61 ± 5.58***	17.17 ± 4.97**	18.68 ± 4.96*	22.08 ± 3.26*^	0.001
90-day good outcome (modified Rankin Score 0–2), n (%)	46(42.6)	12(66.7)a	27(50.9)ab	5(20.0)b	2(16.7)b	0.003
Motality after 90-Day (%)	29(26.9)	1(5.6)	14(26.4)	10(40.0)	4(33.3)	0.05

# Fisher exact probability method. ***Grade 1 versus grade 2/grade 3/grade 4 *P* < 0.05, **Grade 2 versus grade 1/grade 4 *P* < 0.05, *Grade 3 versus grade 1 *P* < 0.05, *^Grade 4 versus grade 1/grade 2 *p* < 0.05. a and b mean significantly different level in each group in outcome(*p* < 0.05). mTICI: modified Thrombolysis in Cerebral Infarction classification; SICH: Symptomatic intracranial hemorrhage; NIHSS, National Institute of Health Stroke Scale; ACA: anterior cerebral artery; PCA: posterior cerebral artery; MCA, middle cerebral artery; ICA, internal carotid artery; mRS, modified Rankin Score.

## Discussion

The circle of Willis serves as the most critical intracranial route for vascularization. In this study, we identified the occluded internal carotid artery and its branches based on the anatomic variation of the circle of Willis and the location and size of the thrombus, and categorized AIICAO patients into four grades. This classification may serve as a predictor for the prognosis of AIICAO patients.

The circle of Willis plays a crucial role in maintaining the stability of cerebral blood flow and perfusion pressure (20–23). In patients with AIICAO, the circle of Willis facilitates the equalization of blood flow from the contralateral hemisphere through the ACoA and PCoA (24, 25). When the ACoA and PCoA are absent or their origin is blocked, the presence of functional Willis collaterals is insufficient to supply the ischemic areas of the brain, potentially leading to more severe clinical outcomes. Based on previous research data, the integrity of Willis was categorized into six types, with which clotting site and size of thrombus, allows for the division of Willis blood flow in iICAO patients can be divided into 17 types (5). All 17 types were further classified into seven categories of Willis compensation, referred to as Model 1, based on the patency of the arteries. This classification effectively distinguishes which arteries were occluded in DSA and provides a superior visual criterion compared to other studies (26, 27). Although the univariate analysis revealed significant differences in etiologic subtype and embolectomy data among the seven groups of Willis compensation, no significant differences were observed in embolectomy data between the pairs of type 1a and 1b, type3 and type 4, type 5 and type 6 groups of Willis compensation (*p* > 0.05). This finding suggests that ACoA and PCoA in the circle of Willis are equally effective in compensating for ischemic brain tissue, while ACA and PCA are similarly capable of supplying ischemic regions of MCA via leptomeningeal collaterals. The conciser Model 2, ranging from grade 1 to grade 4 was developed to evaluate arterial occlusion in AIICAO.

The four grades of the AIICAO represent distinct scenarios of acute cerebral infarction affecting the blood supply to the MCA, ACA and PCA, with each artery extending from the cervical segment to the terminal segment. Additionally, the ipsilateral ACA and PCA may provide leptomeningeal collaterals to the area affected by MCA occlusion sphere. In grade 1 ischemia, brain tissue can be perfused via the ACoA and the ipsilateral PCoA. In the other grades, the MCA can only receive blood supply through the leptomeningeal collaterals of the PCA and ACA. From grade 2 to 4, the extent of ischemic brain tissue gradually increased. At the same time, the leptomeningeal collaterals from the adjacent arteries gradually decreased. Consequently, the NIHSS score upon admission was significantly lower in grade 1 compared to all other grades, with the NIHSS score increasing progressively from grade 2 to grade 4. Grade 1 can receive blood supply from the PCoA and ACoA, while the supplying arteries in the other grades are rogressively diminished, leading to a corresponding reduction in blood supply from the leptomeningeal collaterals. Postoperative mTICI and postoperative sICH are the two primary factors influencing prognosis. The success rate of recanalization was significantly lower in postoperative mTICI grade 4 compared to other grades, and the probability of rebleeding after thrombectomy was also markedly higher in postoperative sICH grades 3 and 4 compared to grades 1 and 2. It was suggested that this might be related to the large occluded blood vessels leading to a higher thrombus burden and a more extensive area of cerebral ischemia. The proportion of favorable 90-day outcomes was significantly lower in types 3 and 4 than in types 1 and 2, with mortality at 90 days exhibiting a similar trend.

In the etiologic subtype of stroke, grades 2 and 3 exhibited a higher proportion of cardioembolism compared to grade 1, which in turn, demonstrated a greater proportion of atherosclerosis *in situ* than grades 2 and 3. The proportion of atherothrombotic plaques in grade 4 was higher than in the other grades. We hypothesized that thrombosis in grade 1 primarily occurs in C5 and C6, where the PCoA, ACA and MCA are uninterrupted. In contrast, thrombosis in grades 2 and 3 is localized to the terminal portion of the ICA (C7), where cardio thrombosis usually occurs. In grade 4, the most common cause is a thrombus that dislodges from the proximal end of the internal carotid artery to the bifurcation, resulting in both acute occlusion of the internal carotid artery and a pathological basis of atherosclerosis, leading to a high NIHSS and difficult thrombectomy, ultimately resulting in a poor prognosis, which is similar to previous study (28).

Assessment of clot location, patency of adjacent arterial segments, and collateral flow to the ischemic territory through angiography can reveal subtle but crucial differences between ICA occlusions. More robust collateral perfusion, accompanied by effective Willis compensation, may facilitate recanalization and predict favorable clinical outcomes and longside a lower mortality rate (29). Conversely, inadequate Willis compensation is associated with a more concerning prognosis. Characterizing terminal ICA occlusions based on the four grades of Willis compensation probably serves as a valuable selection criterion and prognostic tool in future endovascular studies focused on acute ischemic stroke.

The present study has several limitations. First, the sample size is small, which may affect the accuracy of the results. Second, the visualization of the circle of Willis through aortic arch angiography lacks sufficient sufficient clarity. Nevertheless, we used cerebral vascular perfusion imaging and DSA development to elucidate the anatomical structure of the circle of Willis.

## Conclusion

We established and evaluated four grades of Willis compensation base on integrity of the circle of Willis, the size of the thrombus, and the location of the thrombus *in situ*, and this classification probably serve as a valuable prognostic indicator and provide an important reference for screening patients before EVMT.

## Data Availability

The original contributions presented in the study are included in the article/supplementary material, further inquiries can be directed to the corresponding author.
